# Characterization of the bacterial community composition in water of drinking water production and distribution systems in Flanders, Belgium

**DOI:** 10.1002/mbo3.726

**Published:** 2018-10-14

**Authors:** Ado Van Assche, Sam Crauwels, Joseph De Brabanter, Kris A. Willems, Bart Lievens

**Affiliations:** ^1^ Laboratory for Process Microbial Ecology and Bioinspirational Management (PME & BIM) Department of Microbial and Molecular Systems (M2S) KU Leuven, Campus De Nayer Sint‐Katelijne‐Waver Belgium; ^2^ Department of Electrical Engineering (ESAT ‐ STADIUS) KU Leuven Leuven Belgium

**Keywords:** 454‐pyrosequencing, *Acinetobacter*, bacterial community composition, drinking water, next‐generation sequencing

## Abstract

The quality of drinking water is influenced by its chemical and microbial composition which in turn may be affected by the source water and the different processes applied in drinking water purification systems. In this study, we investigated the bacterial diversity in different water samples from the production and distribution chain of thirteen drinking water production and distribution systems from Flanders (Belgium) that use surface water or groundwater as source water. Water samples were collected over two seasons from the source water, the processed drinking water within the production facility and out of the tap in houses along its distribution network. 454‐pyrosequencing of 16S ribosomal RNA gene sequences revealed a total of 1,570 species‐level bacterial operational taxonomic units. Strong differences in community composition were found between processed drinking water samples originating from companies that use surface water and other that use groundwater as source water. *Proteobacteria* was the most abundant phylum in all samples. Yet, several phyla including *Actinobacteria* were significantly more abundant in surface water while *Cyanobacteria* were more abundant in surface water and processed water originating from surface water. *Gallionella*,* Acinetobacter*, and *Pseudomonas* were the three most abundant genera detected. Members of the *Acinetobacter* genus were even found at a relative read abundance of up to 47.5% in processed water samples, indicating a general occurrence of *Acinetobacter* in drinking water (systems).

## 1 INTRODUCTION

The delivery of safe, clean drinking water is important for public health. The quality of the final drinking water is influenced by its chemical and microbial composition. In particular, microbial growth in drinking water can be problematic as it may result in the multiplication and rapid spread of opportunistic pathogens (van der Kooij, Visser, & Hijnen, 1982; LeChevallier, Welch, & Smith, 1996). Additionally, it may lead to aesthetic problems such as deteriorated taste and odor, and technical problems such as corrosion of the pipe material (Camper, 2013; Christensen, Nissen, Arvin., & Albrechtsen, 2011; Hoehn, 1988).

Traditionally, microbiological characterizations of drinking water are specified in national and international norms and rely on culture‐based detection methods such as heterotrophic plate counts and counts of fecal indicator bacteria (i.e., *Escherichia coli*, coliforms, and enterococci) (European Directive 98/83/EG). Although these classical plating methods have greatly helped evaluating the microbial quality of drinking water (Inomata, Chiba, & Hosaka, 2009; Lee & Kim, 2003; Martiny, Albrechtsen, Arvin, & Molin, 2005; September, Els, Venter, & Brozel, 2007), not all microorganisms are culturable under standard laboratory conditions (Byrd, Xu, & Colwell, 1991), by which important species may be overlooked (Liu, Gilchrist, Zhang, & Li, 2008). Culture‐independent DNA‐based methods such as 454 pyrosequencing or Illumina MiSeq sequencing of ribosomal RNA (rRNA) genes overcome these limitations and allow in‐depth analysis of entire microbial community composition with an unprecedented level of resolution (Caporaso et al., 2012; Margulies et al., 2005). Therefore, these technologies are increasingly used to study drinking water microbial community composition and associated biofilms and have greatly contributed to our understanding of the true diversity of these bacterial community compositions (e.g., Hong et al., 2010; Navarro‐Noya et al., 2013; Prest et al., 2014; Roeselers et al., 2015; Wu et al., 2015). Molecular microbial surveys based on 16S rRNA genes in combination with high‐throughput sequencing technologies overcome these constraints, allowing in‐depth analysis of microbial community structures with an unprecedented level of resolution (Hong et al., 2010; Lautenschlager et al., 2013).

Several recent studies have focused on how the microbial community composition in drinking water is shaped by different drinking water production steps and found a significant impact of the treatment method (Lautenschlager et al., 2014; Li et al., 2017; Ma, Vikram, Casson, & Bibby, 2017; Oh, Hammes, & Liu, 2018; Pinto, Xi, & Raskin, 2012; Shaw et al., 2015; Xu, Tang, Ma, & Wang, 2017). Further, recent studies have investigated the spatial and/or long‐term temporal variation in bacterial community composition from source water to tap water (Hull et al., 2017; Pinto, Schroeder, Lunn, Sloan, & Raskin, 2014; Roeselers et al., 2015). Some studies indicated a major impact of seasonal effects on the bacterial community composition (Pinto et al., 2014), while others found the treatment method(s) as most important factor (Ma et al., 2017; Pinto et al., 2012; Roeselers et al., 2015). Nevertheless, still little is known about the impact of the source water on the bacterial community composition in the final drinking water. Therefore, the goal of this study was to assess the bacterial diversity of different water samples from the production and distribution chain of a number of drinking water production and distribution systems (DWPDS) from Flanders (Belgium) that use either surface water (SW) or groundwater (GW) as source water. Additionally, we explored potential differences in the bacterial community composition between two different seasons. Concomitantly, we also identified the most important taxa depending on the type of water and season using an indicator species analysis.

## 2 MATERIAL AND METHODS

### 2.1 Study samples

In total, 41 water samples were collected from 13 DWPDS distributed over Flanders (Belgium). Among these, six DWPDS use SW as their source water, while seven use GW. Six DWPDS were sampled in April 2013 (two using SW; four using GW), six in November 2013 (three using SW; three using GW), and one (using SW) in April and November 2013 (Supporting Information Table S1). For each DWPDS, the source water, the processed water (PW) (immediately taken after the purification process), and the household tap water (HTW) (water delivered to the consumer) were sampled. As a result, samples represented a diverse collection of different water types, including GW, SW, processed water originating from groundwater (PWg), processed water originating from surface water (PWs), household tap water originating from groundwater (HTWg), and household tap water originating from surface water (HTWs). For DWPDS “E6,” the household tap water was not included as this was also supplied with drinking water from another DWPDS (Supporting Information Table S1). Due to confidentiality reasons, information about the water treatment process steps was not provided by the DWPDS surveyed. At each sampling point, after letting running a few liters of water away, 2 L water was collected under aseptic conditions in a sterile bottle, stored in an ice‐cooled container for transport, and further stored at 4°C prior to analysis (maximum within 1 day after sampling).

### 2.2 DNA extraction, PCR amplification, and 454 amplicon pyrosequencing

Following filtration of 2 L water over a 0.45‐μm filter (mixed sterile cellulose ester filter [Millipore, Billerica, MA, USA]), genomic DNA was extracted using the phenol–chloroform extraction method described in Lievens et al. (2003) using the filter as starting material. Obtained DNA was subjected to PCR amplification and 454 pyrosequencing of the 16S rRNA gene. More specifically, an amplicon library was created using the primer combination 515F (5′‐GTGCCAGCMGCCGCGGTAA‐3′) and 806R (5′‐GGACTACVSGGGTATCTAAT‐3′) generating amplicons which cover the prokaryotic (bacterial and archaeal) V4 region of the 16S rRNA gene (Bates et al., 2011). This primer combination has been commonly used in diverse metagenomics studies, including water research (Ng et al., 2015; Wang, Masters, Falkinham, Edwards, & Pruden, 2015; Wu et al., 2015) . “Fusion” primers, required for the 454 pyrosequencing process, were designed according to the guidelines for 454 GS‐FLX Titanium Lib‐L sequencing and contained the Roche 454 pyrosequencing adapters and a sample‐specific multiplex identifier sequence in between the adapter and the forward primer for sample‐specific sequence tracking. A T100 Thermal Cycler (Bio‐Rad, Hercules, CA, USA) was used for PCR amplification. The total reaction volume was 20 μl and contained 1.0 μl 10× diluted genomic DNA, 1.5 μl dNTP mixture (2 mM stock; Invitrogen, Carlsbad, CA, USA), 0.5 μl of each primer (20 μM stock), 2.0 μl 10× Titanium *Taq* PCR buffer, 0.4 μl Titanium *Taq* DNA polymerase (Clontech Laboratories, Palo Alto, CA, USA), and 14.1 μl nuclease‐free water. The following PCR conditions were used as follows: initial denaturation of 2 min at 94°C, followed by 30 cycles of 45 s at 94°C, 45 s at 59°C, and 1 min at 72°C, followed by a final extension phase of 10 min at 72°C. Following agarose gel electrophoresis, amplicons of the expected size range were excised and extracted from the gel using the QIAquick gel extraction kit, according to the manufacturer's instructions (Qiagen, Hilden, Germany). Purified dsDNA amplicons were quantified using a Qubit 2.0 fluorometer and the high‐sensitivity DNA reagent kit (Invitrogen). Next, all samples were diluted to equimolar concentrations and an amplicon library containing 1.00 × 10^9^ molecules/μl per sample was prepared. A final quality check was done on an Agilent Bioanalyzer 2100 with high‐sensitivity chip (Agilent Technologies, Waldbronn, Germany), and the library was sequenced using the Roche GS‐FLX instrument with Titanium chemistry according to manufacturer's instructions (Roche Applied Science, Mannheim, Germany).

Pyrosequencing yielded a total of 1,014,047 reads. Sequences were assigned to the appropriate sample based on their barcodes and primer sequences, allowing zero discrepancies, and were subsequently trimmed from the fusion primer sequence using a custom Python script implemented within the USEARCH v.8 analysis pipeline (Edgar, 2013) (data deposited in the Sequence Read Archive under BioProject accession PRJNA479747 and SRA accession SRP154875). Subsequently, reads with a total expected error threshold above 0.5 for all bases were discarded, so that the most probable number of errors was zero for all sequences that remained in the dataset. Next, remaining sequences (180,562 out of 230,016, after quality filtering) were trimmed to 250 bp and rarefied to the least number of sequences per sample obtained (i.e., 850 sequences per sample). Remaining sequences were then grouped into species‐level operational taxonomic units (OTUs) based on a 3% sequence dissimilarity cutoff while discarding chimeric sequences using the UPARSE greedy algorithm implemented in USEARCH (Edgar, 2013) as well as global singletons (i.e., OTUs representing only a single sequence in the entire dataset) (Brown et al., 2015; Waud, Busschaert, Ruyters, Jacquemyn, & Lievens, 2014). Next, OTUs were assigned taxonomic identities using the “classify.seqs” command in Mothur (v. 1.36.1) (Schloss et al., 2009) using the Silva taxonomy database (Quast et al., 2013). Taxonomic assignments were considered reliable when bootstrap confidence values exceeded 80.

### 2.3 Data analysis

Operational taxonomic unit richness, the Ace richness estimator, Shannon diversity, and Pielou's evenness were calculated using Mothur (v. 1.36.1) (Schloss et al., 2009). Differences in these parameters were assessed using the “aov” function in R (R Development Core Team, 2015). Similarities between the bacterial community composition of the different water types studied (GW, SW, PWg, PWs, HTWg, and HTWs) were quantified using the ANOSIM (ANalysis Of SIMilarities) and ADONIS (i.e., a permutational multivariate analysis of variance using distance matrices) functions of the Vegan package (v. 2.4‐1) (Oksanen, 2013). In both cases, the Bray–Curtis distance matrix (abundance data) was used. The same analyses were performed to assess seasonal effects on the bacterial community composition. Additionally, rarefaction curves, a nonmetric multidimensional scaling (NMDS) plot, and a hierarchically clustered heatmap were created with the Vegan (v. 2.4‐1) and ggplot2 (v. 2.1.1) packages in R. Boxplots were generated using the boxplot function in R. Additionally, an indicator species analysis was performed for each type of water and season using the Indicspecies package (v. 1.7‐1) in R (De Cáceres, 2013; R Development Core Team, 2015). For all samples originating from the same type of source water, core bacteria were determined, that is, OTUs that occurred in at least one sample of the source water, processed water, and tap water. Venn diagrams showing the distribution of the different OTUs over different subgroups were constructed using the VennDiagram package (v. 1.6.19) for R (Chen & Boutros, 2011). Finally, given the fact that a relatively huge proportion of sequences was identified as *Acinetobacter* and that the 16S rRNA gene is known to not vary greatly between *Acinetobacter* species (La Scola, Gundi, Khamis, & Raoult, 2006), OTUs corresponding to the genus *Acinetobacter* were further analyzed in order to improve identification. More specifically, all unique sequences belonging to the *Acinetobacter* OTUs were blasted against a custom database containing the 16S rRNA gene sequences of the type strains of all *Acinetobacter* species with validly published names (at the time of analysis 50 species) and a number of *Acinetobacter* genomic species, that is species that have yet to receive a Latin binomial name but that are genetically different from the formerly described *Acinetobacter* species (Bouvet & Grimont, 1986; Tjernberg & Ursing, 1989). Additionally, to visualize phylogenetic relationships, a maximum‐likelihood phylogenetic tree was constructed based on these sequences using MEGA 5.10 (Kumar, Nei, Dudley, & Tamura, 2008).

## 3 RESULTS

**Figure 1:**
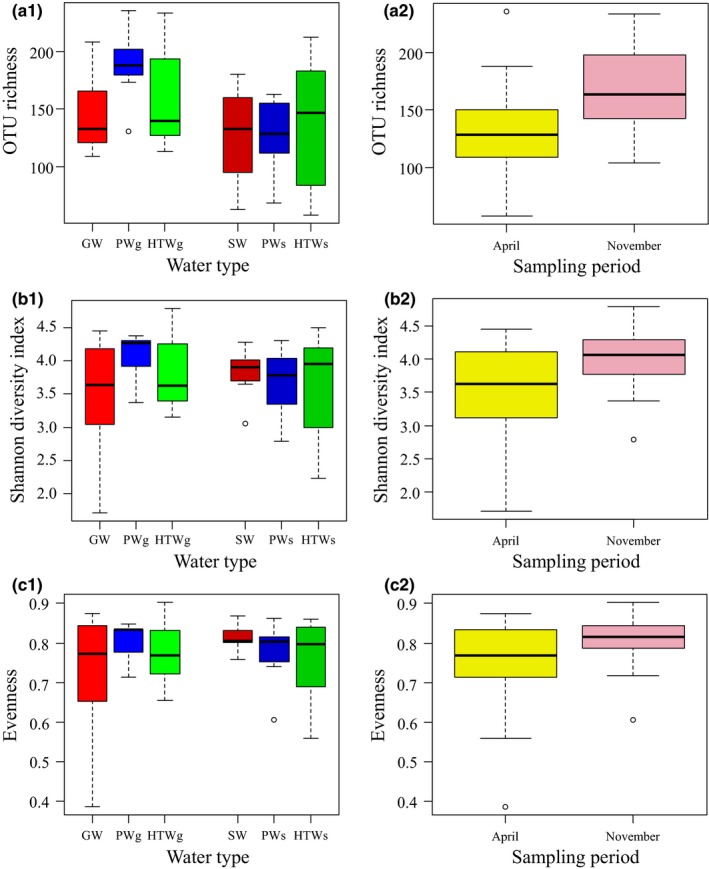
Boxplot representation of OTU richness (a), Shannon diversity (b), and Pielou's evenness (c) of the bacterial communities in the water samples investigated in this study. Water samples were grouped based on water type (a1, b1, and c1) and sampling period (a2, b2, and c2). The boxplots show the upper and lower quartiles; the whiskers indicate variability outside the upper and lower quartiles which is no more than 1.5 times the interquartile range. Further, the median is plotted as a thick black line. GW, groundwater (*n *=* *7); PWg, processed water produced from groundwater (*n *=* *7); HTWg, household tap water processed from groundwater (*n *=* *7); SW, surface water (*n *=* *7); PWs, processed water produced from surface water (*n *=* *7); HTWs, household tap water processed from surface water (*n *=* *6); April (*n *=* *21); November (*n *=* *20)

Following rarefying of all samples to 850 sequences per sample, a total of 1,570 OTUs were recovered, ranging from a minimum of 58 OTUs per sample to a maximum of 235 OTUs per sample (Supporting Information Table S1). Based on the Ace estimator, the mean sampling coverage was 69.4% (range between 50.0% and 100.0%) (Supporting Information Table S1), suggesting that the most abundant bacterial community members were covered, as can also be observed from the rarefaction curves (Supporting Information Figure S1). No significant differences (*p *<* *0.05) could be observed between the number of OTUs per sample between the different water types (groundwater, surface water, processed water originated from groundwater or surface water, and household tap water originated from groundwater or surface water) (Figure 1; Supporting Information Table S2). Likewise, no significant differences were found in the calculated diversity indices (Figure 1; Supporting Information Table S2). By contrast, significant differences in OTU richness, Ace, and Shannon diversity were observed between the two sampling periods (i.e., April and November; Figure 1; Supporting Information Table S2), but not for the evenness (*p *>* *0.05). Significant differences (*p *<* *0.05) were also found when the communities of the different water types were analyzed using ANOSIM and ADONIS (Table 1). Greatest differences were observed between the microbial community composition from surface versus groundwater (*p *<* *0.001 for both ANOSIM and ADONIS), and the least differences were observed between the bacterial community composition of HTWg versus HTWs (*p *=* *0.069 and 0.040 for ANOSIM and ADONIS, respectively; Table 1). When seasonal effects were evaluated, no substantial differences were observed within the different water types (*p* value ranging from 0.109 to 0.811 for ANOSIM, and from 0.069 to 0.500 for ADONIS; Table 1), except for the surface water and the PWs (*p *≤* *0.05; Table 1).

**Table 1:** Values and significance scores of the ANOSIM and ADONIS functions

Grouping of samples	ANOSIM		ADONIS	
	*R*	*p* Value	*F*	*p* Value
Overall comparison based on the different water types^a^ (i.e., GW, SW, PW [g & s], and HTW [g & s])	0.356	0.001***	1.970	0.001***
Comparison of the different water types based on the source of the source water (i.e., groundwater vs. surface water)				
Source water	0.643	0.001***	3.589	0.001***
Processed water	0.390	0.002**	1.734	0.036*
Household tap water	0.177	0.069	1.419	0.040*
Comparison of the different sampling periods (i.e., April vs. November)				
Groundwater	0.232	0.109	1.292	0.100.
Surface water	0.694	0.026*	1.994	0.036*
Processed water (produced from GW)	0.185	0.144	1.255	0.069.
Processed water (produced from SW)	0.676	0.050*	2.053	0.032*
Household tap water (produced from GW)	−0.157	0.811	1.062	0.335
Household tap water (produced from SW)	−0.037	0.500	0.917	0.500

a Different water types: GW, groundwater; SW, surface water; PW, processed water; and HTW, household tap water; g or s, originating from groundwater or surface water, respectively. Significance levels: 0 ‘***’ 0.001 ‘**’ 0.01 ‘*’ 0.05

**Figure 2:**
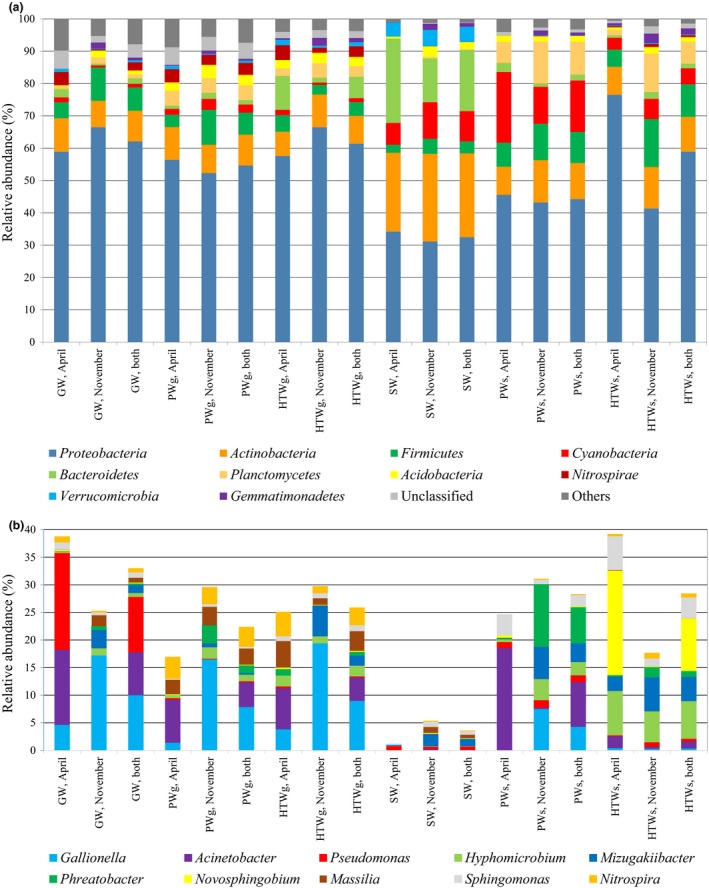
(a) Relative abundance of bacterial phyla in the different water samples collected in April, November, and both sampling periods combined. Phyla representing <5% of the sequences (in total) are grouped together as “Others.” (b) Relative abundance of the 10 most abundant genera in the different water samples collected in April, November, and both sampling periods combined. Numbers of samples included are reported between brackets. GW, groundwater (*n *=* *7); PWg, processed water produced from groundwater (*n *=* *7); HTWg, household tap water processed from groundwater (*n *=* *7); SW, surface water (*n *=* *7); PWs, processed water produced from surface water (*n *=* *7); HTWs, household tap water processed from surface water (*n *=* *6)

Taxonomic assignment of the OTUs revealed the presence of 28 bacterial and archaeal phyla and 253 genera (Supporting Information Table S3) with an officially published scientific name. *Proteobacteria* was the most abundant phylum detected (52.1% of the total number of sequences), followed by *Actinobacteria* (12.6%) and *Firmicutes* (6.9%). Based on water type, analysis of variance indicated a significantly higher relative abundance of the phyla *Actinobacteria*,* Bacteroidetes*, and *Verrucomicrobia* in the surface water (*p *<* *0.05). Further, members of the phylum *Cyanobacteria* were more abundantly present in surface water and PWs (Figure 2). Furthermore, relative abundance of the phyla *Firmicutes* and *Gemmatimonadetes* was higher in November than in April (*p *<* *0.05). Analysis of variance also indicated a higher relative abundance of *Nitrospirae* in water samples from facilities using groundwater (*p *<* *0.05) (Figure S2, Supporting Information). Indeed, highest number of *Nitrospirae* sequences were observed in two production systems located in the province of Antwerp using groundwater (A1 and A2).

**Figure 3:**
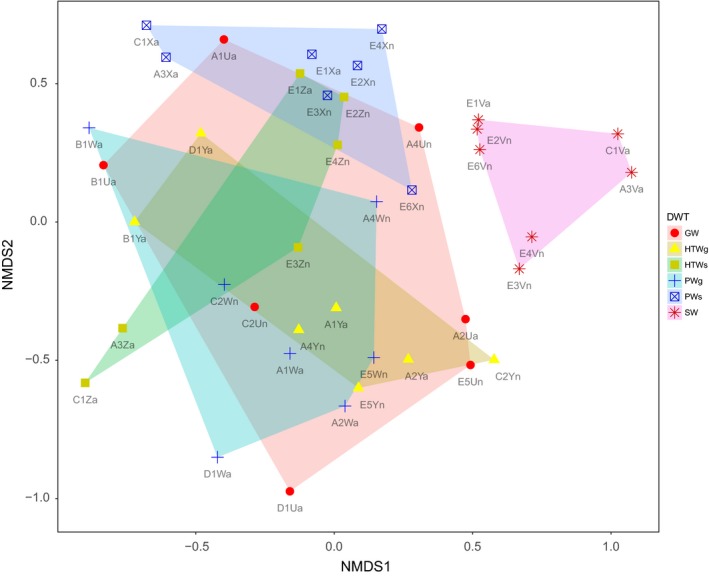
Nonmetric dimensional scaling (NMDS) ordination plot of the bacterial community composition (stress value 0.242) of all water samples studied (based on Bray–Curtis distance matrix (abundance data)). GW, groundwater; PWg, processed water originating from groundwater; HTWg, household tap water originating from groundwater; SW, surface water; PWs, processed water originating from surface water; HTWs, household tap water originating from surface water. For more information about the studied samples, the reader is referred to Supporting Information Table S1

**Figure 4:**
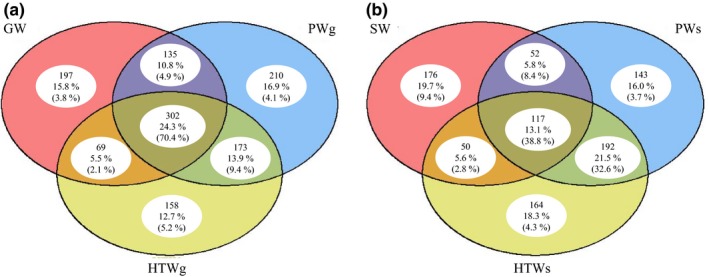
Venn diagrams illustrating the OTU distribution over different water types, including water samples related to production systems using groundwater as source water (GW (*n *=* *7), PWg (*n *=* *7) and HTWg (*n *=* *7)) (a) and water samples related to production systems using surface water as source water (SW (*n *=* *7), PWs (*n *=* *7) and HTWs (*n *=* *6)) (b). When an OTU occurred in at least one sample of each of the subgroups of water types, it was put in the intersection of the groups. The numbers within the Venn diagrams represent: top, number of OTUs within the subset; middle: percentage of OTUs representing the number of OTUs within the subgroup; and bottom: percentage of sequences representing the OTUs within the subgroup. GW, groundwater; PWg, processed water originating from groundwater; HTWg, household tap water originating from groundwater; SW, surface water; PWs, processed water originating from surface water; HTWs, household tap water originating from surface water

When zooming in at genus level, the 10 most abundant genera encountered in this study encompassed the genera *Gallionella* (5.4% of all sequences recovered), *Acinetobacter* (4.4%), *Pseudomonas* (2.2%), *Hyphomicrobium* (2.1%), *Mizugakiibacter* (2.1%), *Phreatobacter* (1.7%), *Novosphingobium* (1.5%), *Massilia* (1.4%), *Sphingomonas* (1.4%), and *Nitrospira* (1.4%) (Figure 2). Whereas these genera were generally found in the different water types investigated, *Gallionella* and *Phreatobacter* species were not detected in any sample from the surface water. The NMDS ordination of the bacterial community composition revealed a clear clustering of the surface water samples, while samples from the other water types appeared scattered on the plot (Figure 3), as can also be observed from the heatmap clustering shown in Supporting Information Figure S3. The clustering of the different surface water samples indicates that the bacterial community composition of surface water is more similar to each other than to water samples of another origin and is characterized by a specific microbial community composition. Indeed, indicator species analysis revealed as much as 63 OTUs as significant indicators for the surface water bacterial community composition (Supporting Information Table S4). Most of these OTUs represented taxa belonging to the phylum of *Actinobacteria* (Supporting Information Table S4). Furthermore, indicator species analysis revealed the presence of a number of unique OTUs within particular DWPDS (i.e., for DWPDS A2, B1, D1, and E5, indicating that these DWPDS are characterized by particular bacterial populations (data not shown). Indicator species analysis also revealed 18 and 70 indicator OTUs for April and November, respectively (Supporting Information Table S5). In order to evaluate differences in core OTUs and the OTU distribution between the samples originating from groundwater and those from surface water, a Venn diagram was generated (Figure 4). In total, 1,244 and 894 OTUs out of the 1,570 OTUs were present in the subgroup containing the groundwater‐derived samples and the subgroup containing the surface water‐derived samples, respectively. For the first set, a core community of 302 bacterial OTUs was observed, representing 24.3% and 70.4% of the OTUs and sequences, respectively. For the surface water‐related samples, the core community consisted of 117 OTUs, representing 13.1% and 38.8% of the OTUs and sequences, respectively. Overall the core community of groundwater‐related samples was represented by 18 different bacterial phyla, while the core community of surface water‐related samples was represented by 13 phyla. In both cases, *Proteobacteria* was the most abundant phylum corresponding to 68.3% and 52.2% of the core community sequences for groundwater‐ and surface water‐related samples, respectively. Further, the core community of the groundwater‐related samples mainly consisted of *Actinobacteria* (10.1%), *Firmicutes* (6.9%), *Nitrospirae* (3.9%), and *Acidobacteria* (1.8%), together with the *Proteobacteria* covering over 90% of the core community sequences. For the surface water‐related core community, aside from *Proteobacteria* (52.2%), the majority of sequences belonged to *Actinobacteria* (18.9%), *Firmicutes* (10.4%), *Cyanobacteria* (4.8%), and *Bacteroidetes* (4.4%). Phyla and candidate phyla which were found in groundwater‐related samples but not in surface water‐related samples were Candidate division OP3, *Omnitrophica*, SHA‐109, *Parcubacteria*, and *Thaumarchaeota*. The candidate phylum WD272 was present in surface water‐related samples but not in groundwater‐related samples.

In general, members of the *Acinetobacter* genus were abundantly found in the water samples studied, reaching read abundances of up to 47.5% for the groundwater sample B1Ua. More particularly, *Acinetobacter* was the most abundant bacterium in several processed water samples taken in April (A3Xa, B1Wa, C1Xa, and E1Xa). Additionally, it was also the most abundant genus in the groundwater sample B1Ua and sample D1Ya, a household tap water sample taken in April (Supporting Information Table S3). Strikingly, whereas *Acinetobacter* was abundantly present in the processed water samples of April, the bacterium was not detected in the corresponding surface water samples (Figure 2; Supporting Information Table S3). In total, three OTUs were associated with *Acinetobacter* (OTU 1, 293, and 1434; Supporting Information Table S3). OTU 1, which represented the most abundant OTU in this study (4.34% of all sequences studied), was found in all water types investigated with the exception of surface water (Supporting Information Table S3). In contrast, OTU 293 was not detected in processed and HTWg and OTU 1434 was not present in surface water neither in groundwater. When comparing and positioning the unique sequences of each of these OTUs in a phylogenetic tree containing the 16S rRNA gene sequences of the type strains of all known *Acinetobacter* species as well as a number of *Acinetobacter* genomic species, most OTU 1 sequences showed highest homology with *A. calcoaceticus*,* A. pitti*,* A. nosocomialis*,* A. seifertii*,* A. dijkshoorniae*, and the genomic species “between 1 and 3,” whereas a few sequences clustered a bit further away (Supporting Information Figure S4). Most of the unique sequences of OTU 293 and OTU 1434 clustered closely with the type strain of *A. johnsonii*. For OTU 1434, a number of sequences showed highest homology with *A. baumannii*, known as an opportunistic pathogen in humans (Antunes, Visca, & Towner, 2014; Dijkshoorn, Nemec, & Seifert, 2007) (Supporting Information Figure S4). Further, a number of sequences were found clustering together with other *Acinetobacter* species (Supporting Information Figure S4), suggesting that in total, many *Acinetobacter* species were found in the water samples investigated in this study.

## 4 DISCUSSION

In order to support drinking water quality, there is a strong interest in the microbial community composition of drinking water and how the community changes depending on the source water, from the source water to the household tap water, and during the season. Whereas drinking water microbial community compositions have been classically studied using plating techniques, here, 454 amplicon pyrosequencing was used to investigate these questions.

In line with other studies (Pinto et al., 2012; Prest et al., 2014; Wu et al., 2015) phyla such as *Proteobacteria*,* Actinobacteria*,* Firmicutes*,* Cyanobacteria*,* Bacteroidetes*, and *Nitrospirae* were commonly found in the water samples investigated. As also observed in this study, several studies have identified *Proteobacteria* as the most abundant phylum in aquatic environments within the drinking water production industry (Bautista‐de los Santos et al., 2016; El‐Chakhtoura et al., 2015; Liu et al., 2014; Vaz‐Moreira, Nunes, & Manaia, 2017; Zanacic, McMartin, & Stavrinides, 2017). It is clear from our results that the bacterial community composition of surface water strongly differs from those of the other water types studied. Indeed, members of the phyla *Actinobacteria*,* Bacteroidetes*, and *Verrumicrobia* were significantly more abundant in the surface water samples. Additionally, members of the phylum *Cyanobacteria* were abundantly present in surface water and PWs. Moreover, a huge number of OTUs could be identified as a robust indicator for surface water bacterial community composition, including (among several others) several OTUs belonging to the *Actinobacteria*. These observations were also confirmed by the core community analysis of groundwater‐ and surface water‐related subcategories.

Interestingly, whereas significant differences in the bacterial community composition could be observed based on the source of the water at the early stages of the drinking water production and distribution chain, no major differences were found at the stage of the tap, indicating that in general water with a similar microbial composition is delivered irrespective of the water source (Henne, Kahlisch, Höfle, & Brettar, 2013; Pinto et al., 2012; Roeselers et al., 2015). A similar conclusion can be drawn when also different sampling periods were taken into account. Further, comparison of the two sampling periods indicated that especially, the *Firmicutes* and *Gemmatimondetes* were more abundantly present in water samples of November versus April. Moreover, in total, species richness was found to be higher in November than in April. Nevertheless, significant differences based on the ANOSIM and ADONIS functions were only confirmed for samples from the surface water or PWs, reinforcing that seasonal changes have less impact on the bacterial community composition of water of DWPDS which use groundwater as source water instead of surface water. A main limitation of the current study is that only a limited set of samples was investigated. Therefore, in order to draw strong conclusions on how the bacterial community composition is influenced by the source of the water as well as by seasonal influences, further investigation is needed using more samples from different DWPDS sampled over a longer period of time. Further, it is reasonable to assume that also the different treatment steps applied within the different companies may have influenced the dynamics of the microbial community composition along the distribution system (Shaw et al., 2015; Xu et al., 2017).

Analyses performed at the genus level revealed the common presence of well‐known aquatic bacterial genera such as *Gallionella*,* Acinetobacter*,* Pseudomonas*,* Novosphingobium*,* Nitrospira*,* Massilia*,* Sphingomonas*, and *Flavobacterium* (Allen, Edberg, & Reasoner, 2004; Berry, Xi, & Raskin, 2006; Gallego, Sánchez‐Porro, García, & Ventosa, 2006). The relatively newly described genera *Mizugakiibacter* and *Phreatobacter* completed the top 10 of most commonly found genera in this study. *Mizugakiibacter* was recently isolated and described from a sediment sample from a freshwater lake and contains one species to date (i.e., *Muzigakiibacter sediminis*, Kojima, Tokizawa, & Fukui, 2014). Also *Phreatobacter* has been recently described as a novel genus based on a number of strains isolated from ultrapure water of a Hungarian power plant, and currently, one species has been described within the genus (*Phreatobacter oligotrophus*, Tóth et al., 2014). Interestingly, *Acinetobacter* was one of the most abundant taxa encountered in this study, especially in April. In total, three *Acinetobacter* OTUs were identified, among which OTU 1, representing 4.34% of all sequences recovered, was found to be a good indicator for samples taken in April. *Acinetobacter* are aerobic, nonmotile, gram‐negative bacteria that are ubiquitous in the environment and have been identified in drinking water, sewage water, groundwater, dental lines, rivers, soil, human skin, vegetables, flowers and fruits, ponds, and swamps (Álvarez‐Pérez, Lievens, Jacquemyn, & Herrera, 2013; Barbeau et al., 1996; Baumann, 1968; Doughari, Ndakidemi, Human, & Benade, 2011; Guardabassi, Dalsgaard, & Olsen, 1999; Van Assche et al., 2017). Although *Acinetobacter* are not generally considered pathogenic, the *A. baumannii*–*A. calcoaceticus* complex is increasingly associated with nosocomial infections in compromised patients. *Acinetobacter* have been associated with several kinds of infections including respiratory infections, wound infections, bacteremia, secondary meningitis, and urinary infections (Dijkshoorn et al., 2007; Doughari et al., 2011; Visca, Seifert, & Towner, 2011). In immunocompromised patients mortality rates can be as high as 64% (García‐Garmendia et al., 2001), especially because many *Acinetobacter* strains are multidrug resistant (Narciso‐da‐Rocha, Vaz‐Moreira, Svensson‐Stadler, Moore, & Manaia, 2013). Therefore, the presence of *Acinetobacter* in drinking water requires a high level of alertness (Zhang et al., 2013). Phylogenetic analysis revealed that the *Acinetobacter* sequences retrieved in this study were closely related to multiple *Acinetobacter* spp., including the most clinically important species, that is, *A. baumannii*. Therefore, future studies should focus on the isolation and further characterization (both genetically and phenotypically) of these drinking water‐associated acinetobacters, as well as on their clinical relevance in order to better understand the true relevance of this genus for the DWPDS industry.

## CONFLICT OF INTEREST

All authors declare that they have no conflict of interests.

## AUTHORS CONTRIBUTION

A.V.A., K.A.W., and B.L. conceived the ideas and designed methodology. A.V.A. collected the data. A.V.A., S.C., J.D.B., and B.L. analyzed the data. A.V.A. and B.L. led the writing of the manuscript. All authors contributed critically to the drafts and gave final approval for publication. The authors have declared that no competing interests exist.

## ETHICS STATEMENT

This article does not contain any studies with human participants or animals performed by any of the authors.

## Data Availability

The pyrosequencing data are deposited in the Sequence Read Archive under BioProject accession PRJNA479747 and SRA accession SRP154875.
