# Supplementary material for: Characterization of the bacterial community composition in water of drinking water production and distribution systems in Flanders, Belgium
Source: Microbiologyopen. 2018 Oct 14;8(5):e00726. doi: 10.1002/mbo3.726 (PMC6528567; doi:10.1002/mbo3.726)
Supplement: Supplementary file 1 [file MBO3-8-e00726-s001.docx]

**Characterization of the bacterial community composition in water of drinking water production and distribution systems in Flanders, Belgium**

**Ado Van Assche^1^, Sam Crauwels^1^, Joseph De Brabanter^2^, Kris A. Willems^1^, Bart Lievens^1,*^**

*^1^ Laboratory for Process Microbial Ecology and Bioinspirational Management (PME&BIM), Department of Microbial and Molecular Systems (M2S), KU Leuven, Campus De Nayer, B-2860 Sint-Katelijne-Waver, Belgium*

*^2^ Department of Electrical Engineering (ESAT - STADIUS), KU Leuven, B-3001 Leuven, Belgium*

^*^ Corresponding author at: Laboratory for Process Microbial Ecology and Bioinspirational Management (PME&BIM), Department of Microbial and Molecular Systems (M2S), KU Leuven, Campus De Nayer, Fortsesteenweg 30A, B-2860 Sint-Katelijne-Waver, Belgium.

Phone: +32 15 305590. Fax: +32 15 305599. E-mail address: [bart.lievens@kuleuven.be](mailto:bart.lievens@kuleuven.be) (B. Lievens).

**Supplementary information**

**Table S1:** Bacterial community diversity indices for the different water samples investigated in this study.

| **Sample identifier^a^** | **Geographical**  **origin** | **Water type^b^** | **Sampling period** | **Sobs^c^** | **Ace^d^** | **Coverage [%]^e^** | **Shannon^f^** | **Evenness^g^** |
| --- | --- | --- | --- | --- | --- | --- | --- | --- |
| A1Ua | Antwerp | GW | April | 150 | 170.2 | 88.1 | 4.3 | 0.866 |
| A1Wa | Antwerp | PWg | April | 186 | 282.5 | 65.8 | 4.4 | 0.835 |
| A1Ya | Antwerp | HTWg | April | 140 | 259.7 | 53.9 | 3.6 | 0.716 |
| A2Ua | Antwerp | GW | April | 181 | 270.0 | 67.0 | 4.6 | 0.875 |
| A2Wa | Antwerp | PWg | April | 235 | 437.0 | 53.8 | 4.6 | 0.833 |
| A2Ya | Antwerp | HTWg | April | 183 | 283.8 | 64.5 | 4.3 | 0.818 |
| A3Va | Antwerp | SW | April | 63 | 77.4 | 81.4 | 3.1 | 0.758 |
| A3Xa | Antwerp | PWs | April | 129 | 150.4 | 85.8 | 3.9 | 0.803 |
| A3Za | Antwerp | HTWs | April | 84 | 116.8 | 71.9 | 3.1 | 0.689 |
| A4Un | Antwerp | GW | November | 122 | 180.5 | 67.6 | 3.7 | 0.773 |
| A4Wn | Antwerp | PWg | November | 192 | 361.1 | 53.2 | 4.3 | 0.817 |
| A4Yn | Antwerp | HTWg | November | 204 | 296.2 | 68.9 | 4.5 | 0.847 |
| B1Ua | Limburg | GW | April | 120 | 138.6 | 86.6 | 2.9 | 0.586 |
| B1Wa | Limburg | PWg | April | 131 | 159.7 | 82.0 | 3.5 | 0.713 |
| B1Ya | Limburg | HTWg | April | 123 | 123.0 | 100.0 | 3.7 | 0.769 |
| C1Va | East Flanders | SW | April | 68 | 68.0 | 100.0 | 3.7 | 0.869 |
| C1Xa | East Flanders | PWs | April | 69 | 70.5 | 97.9 | 3.1 | 0.741 |
| C1Za | East Flanders | HTWs | April | 58 | 84.1 | 69.0 | 2.3 | 0.558 |
| C2Un | East Flanders | GW | November | 208 | 416.1 | 50.0 | 4.4 | 0.820 |
| C2Wn | East Flanders | PWg | November | 173 | 285.3 | 60.6 | 3.8 | 0.735 |
| C2Yn | East Flanders | HTWg | November | 113 | 159.2 | 71.0 | 3.5 | 0.728 |
| D1Ua | Flemish Brabant | GW | April | 109 | 216.9 | 50.3 | 2.0 | 0.385 |
| D1Wa | Flemish Brabant | PWg | April | 188 | 255.5 | 73.6 | 4.5 | 0.847 |
| D1Ya | Flemish Brabant | HTWg | April | 132 | 176.9 | 74.6 | 3.2 | 0.655 |
| E1Va | West Flanders | SW | April | 123 | 186.0 | 66.1 | 3.8 | 0.797 |
| E1Xa | West Flanders | PWs | April | 120 | 196.0 | 61.2 | 3.6 | 0.762 |
| E1Za | West Flanders | HTWs | April | 130 | 189.3 | 68.7 | 3.9 | 0.794 |
| E2Vn | West Flanders | SW | November | 133 | 191.5 | 69.5 | 4.0 | 0.812 |
| E2Xn | West Flanders | PWs | November | 104 | 157.1 | 66.2 | 2.8 | 0.606 |
| E2Zn | West Flanders | HTWs | November | 163 | 238.3 | 68.4 | 4.3 | 0.839 |
| E3Vn | West Flanders | SW | November | 156 | 254.8 | 61.2 | 4.1 | 0.806 |
| E3Xn | West Flanders | PWs | November | 163 | 252.1 | 64.7 | 4.4 | 0.863 |
| E3Zn | West Flanders | HTWs | November | 212 | 339.5 | 62.4 | 4.6 | 0.860 |
| E4Vn | West Flanders | SW | November | 180 | 290.9 | 61.9 | 4.4 | 0.850 |
| E4Xn | West Flanders | PWs | November | 152 | 190.2 | 79.9 | 4.1 | 0.817 |
| E4Zn | West Flanders | HTWs | November | 183 | 278.9 | 65.6 | 4.2 | 0.801 |
| E5Un | West Flanders | GW | November | 133 | 247.0 | 53.8 | 3.5 | 0.718 |
| E5Wn | West Flanders | PWg | November | 212 | 387.9 | 54.7 | 4.5 | 0.833 |
| E5Yn | West Flanders | HTWg | November | 233 | 385.4 | 60.5 | 4.9 | 0.902 |
| E6Vn | West Flanders | SW | November | 164 | 241.6 | 67.9 | 4.1 | 0.806 |
| E6Xn | West Flanders | PWs | November | 158 | 206.9 | 76.4 | 4.1 | 0.812 |

^a^ Sample identifiers “(A-E)(1-6)(U-Z)(a or n)” contain information about their origin: A-E: geographical origin (A, Antwerp; B, Limburg; C, East Flanders; D, Flemish Brabant; E, West Flanders); 1-6: studied company within a particular region; U-Z: water type (U, groundwater; V, surface water; W, processed water produced from groundwater; X, processed water produced from surface water; Y, household tap water processed from groundwater; Z, household tap water processed from surface water); a, April; n, November.

^b^ GW, groundwater; PWg, processed water produced from groundwater; HTWg, household tap water processed from groundwater; SW, surface water; PWs, processed water produced from surface water; HTWs, household tap water processed from surface water.

^c^ Observed richness.

^d^ Abundance-based coverage estimator.

^e^ Observed richness/Ace estimate * 100.

^f^ Shannon-Wiener diversity index.

^g^ Peilou’s evenness: entropy (Shannon-Wiener diversity index) divided by the logarithm of the number of OTUs.

**Table S2:** Analysis of variance (ANOVA) of the bacterial community diversity indices.

| **Grouping of samples** | **Number of OTUs** | | **Ace^b^** | | **Shannon^c^** | | **Evenness^d^** | |
| --- | --- | --- | --- | --- | --- | --- | --- | --- |
|  | F value | *p* value | F value | *p* value | F value | *p* value | F value | *p* values |
| Water type^a^ (i.e. GW, SW, PWg, PWs, HTWg and HTWg) | 2.098 | 0.089 | 2.171 | 0.080 | 0.722 | 0.612 | 0.758 | 0.568 |
| Sampling period (i.e. April and November) | 8.642 | 0.006 | 9.576 | 0.004 | 6.738 | 0.013 | 3.381 | 0.074 |

^a^ Different water types: GW, groundwater; SW, surface water; PW, processed water; and HTW, household tap water; g or s, originating from groundwater or surface water, respectively.

^b^  Abundance-based coverage estimator.

^c^ Shannon-Wiener diversity index.

^d^ Peilou’s evenness: entropy (Shannon-Wiener diversity index) divided by the logarithm of the number of OTUs.

**Table S3:** Identification^a^ of operational taxonomic units (OTUs) according to the Silva database and distribution over the investigated samples.

^a^ Taxonomic assignment scores are provided between brackets. In general, taxonomic assignments are considered reliable when bootstrap confidence values exceed > 80.

See excel file: Van Assche et al_TableS3_taxonomy.xlsx

Table S4: Results of the indicator species analysis for the different water types studied.

| **Water type^a^** | **OTU ID** | **Phylum^b^** | **Genus^c^** | **A^d^** | **B^e^** | ***p* value^f^** |
| --- | --- | --- | --- | --- | --- | --- |
| GW | 820 | ***Proteobacteria* (100)** | unclassified | 1.000 | 0.571 | 0.005 |
|  | 448 | ***Proteobacteria* (100)** | unclassified | 0.769 | 0.571 | 0.009 |
|  | 473 | ***Microgenomates* (91)** | unclassified | 1.000 | 0.429 | 0.019 |
|  | 426 | ***Proteobacteria* (100)** | unclassified | 1.000 | 0.429 | 0.019 |
|  | 378 | ***Firmicutes* (100)** | unclassified | 0.698 | 0.571 | 0.023 |
|  | 66 | ***Nitrospirae* (86)** | Candidatus Magnetoovum (50) | 0.927 | 0.429 | 0.036 |
|  | 957 | ***Proteobacteria* (100)** | unclassified | 0.800 | 0.429 | 0.050 |
|  | 1317 | Candidate division OP3(94) | unclassified | 0.800 | 0.429 | 0.037 |
|  |  |  |  |  |  |  |
| PWg | 139 | ***Proteobacteria* (100)** | ***Variibacter* (85)** | 0.901 | 0.714 | 0.027 |
|  | 864 | ***Proteobacteria* (100)** | unclassified | 1.000 | 0.571 | 0.001 |
|  | 801 | Candidate division OP3 (29) | unclassified | 0.929 | 0.571 | 0.010 |
|  | 51 | ***Proteobacteria* (100)** | unclassified | 0.788 | 0.571 | 0.016 |
|  | 446 | *Gracilibacteria* (56) | unclassified | 1.000 | 0.429 | 0.021 |
|  | 781 | ***Proteobacteria* (99)** | unclassified | 1.000 | 0.429 | 0.019 |
|  | 1159 | ***Proteobacteria* (100)** | ***Legionella* (100)** | 1.000 | 0.429 | 0.021 |
|  | 597 | ***Parcubacteria* (99)** | unclassified | 0.600 | 0.571 | 0.045 |
|  |  |  |  |  |  |  |
| HTWg | 707 | ***Gemmatimonadetes* (100)** | unclassified | 1.000 | 0.429 | 0.022 |
|  |  |  |  |  |  |  |
| SW | 93 | ***Actinobacteria* (100)** | hgcI clade (89) | 0.968 | 1.000 | 0.001 |
|  | 56 | ***Actinobacteria* (100)** | hgcI clade (51) | 0.943 | 1.000 | 0.001 |
|  | 21 | ***Actinobacteria* (100)** | Candidatus Limnoluna (92) | 0.934 | 1.000 | 0.001 |
|  | 98 | ***Proteobacteria* (100)** | ***Polynucleobacter* (100)** | 0.914 | 1.000 | 0.001 |
|  | 37 | ***Actinobacteria* (100)** | hgcI clade (100) | 0.902 | 1.000 | 0.001 |
|  | 32 | ***Actinobacteria* (100)** | hgcI clade (100) | 0.887 | 1.000 | 0.001 |
|  | 121 | ***Bacteroidetes* (100)** | unclassified | 0.859 | 1.000 | 0.001 |
|  | 191 | ***Actinobacteria* (100)** | Candidatus Rhodoluna (99) | 0.857 | 1.000 | 0.001 |
|  | 148 | ***Actinobacteria* (100)** | CL500-29 marine group (100) | 1.000 | 0.857 | 0.001 |
|  | 365 | ***Bacteroidetes* (100)** | ***Fluviicola* (93)** | 1.000 | 0.857 | 0.001 |
|  | 349 | ***Proteobacteria* (100)** | ***Arenimonas* (99)** | 1.000 | 0.857 | 0.001 |
|  | 25 | ***Bacteroidetes* (100)** | ***Fluviicola* (100)** | 0.971 | 0.857 | 0.001 |
|  | 205 | ***Actinobacteria* (100)** | ***Alpinimonas* (98)** | 0.968 | 0.857 | 0.001 |
|  | 212 | ***Proteobacteria* (100)** | LD28 freshwater group (100) | 0.954 | 0.857 | 0.001 |
|  | 95 | ***Actinobacteria* (100)** | hgcI clade (94) | 0.952 | 0.857 | 0.001 |
|  | 76 | ***Verrucomicrobia* (100)** | unclassified | 0.933 | 0.857 | 0.001 |
|  | 40 | ***Cyanobacteria* (100)** | unclassified | 0.930 | 0.857 | 0.001 |
|  | 71 | ***Bacteroidetes* (100)** | ***Pseudarcicella* (100)** | 0.929 | 0.857 | 0.001 |
|  | 335 | ***Actinobacteria* (100)** | hgcI clade (60) | 0.889 | 0.857 | 0.001 |
|  | 102 | ***Bacteroidetes* (100)** | ***Algoriphagus* (96)** | 1.000 | 0.714 | 0.001 |
|  | 242 | ***Actinobacteria* (100)** | hgcI clade (99) | 1.000 | 0.714 | 0.001 |
|  | 58 | ***Bacteroidetes* (100)** | ***Sediminibacterium* (99)** | 0.706 | 1.000 | 0.001 |
|  | 209 | ***Verrucomicrobia* (100)** | unclassified | 0.975 | 0.714 | 0.001 |
|  | 36 | ***Proteobacteria* (100)** | ***Polaromonas* (92)** | 0.673 | 1.000 | 0.002 |
|  | 134 | ***Actinobacteria* (100)** | Candidatus Planktoluna (96) | 0.939 | 0.714 | 0.001 |
|  | 967 | ***Bacteroidetes* (100)** | ***Flavobacterium* (100)** | 0.777 | 0.857 | 0.001 |
|  | 34 | ***Bacteroidetes* (100)** | ***Flavobacterium* (100)** | 0.922 | 0.714 | 0.001 |
|  | 1172 | ***Proteobacteria* (100)** | *Simplicispira* (61) | 0.767 | 0.857 | 0.002 |
|  | 27 | ***Bacteroidetes* (100)** | ***Flavobacterium* (100)** | 0.884 | 0.714 | 0.002 |
|  | 87 | ***Actinobacteria* (100)** | hgcI clade (100) | 0.875 | 0.714 | 0.001 |
|  | 185 | ***Bacteroidetes* (100)** | *Flavisolibacter* (43) | 0.875 | 0.714 | 0.001 |
|  | 990 | ***Bacteroidetes* (100)** | ***Flavobacterium* (100)** | 0.862 | 0.714 | 0.002 |
|  | 11 | ***Proteobacteria* (100)** | unclassified | 0.857 | 0.714 | 0.003 |
|  | 179 | ***Bacteroidetes* (100)** | ***Fluviicola* (98)** | 0.825 | 0.714 | 0.005 |
|  | 269 | ***Verrucomicrobia* (100)** | unclassified | 1.000 | 0.571 | 0.001 |
|  | 538 | ***Proteobacteria* (100)** | unclassified | 1.000 | 0.571 | 0.003 |
|  | 308 | ***Bacteroidetes* (100)** | ***Leadbetterella* (100)** | 1.000 | 0.571 | 0.001 |
|  | 450 | ***Bacteroidetes* (100)** | unclassified | 1.000 | 0.571 | 0.001 |
|  | 373 | ***Verrucomicrobia* (100)** | *Haloferula* (50) | 1.000 | 0.571 | 0.001 |
|  | 454 | ***Verrucomicrobia* (100)** | ***Prosthecobacter* (100)** | 0.928 | 0.571 | 0.006 |
|  | 400 | ***Bacteroidetes* (100)** | *Flavisolibacter* (68) | 0.905 | 0.571 | 0.003 |
|  | 598 | ***Bacteroidetes* (100)** | unclassified | 0.900 | 0.571 | 0.002 |
|  | 773 | ***Proteobacteria* (100)** | ***Aeromonas* (100)** | 0.875 | 0.571 | 0.004 |
|  | 414 | ***Bacteroidetes* (100)** | ***Fluviicola* (100)** | 0.833 | 0.571 | 0.009 |
|  | 1394 | ***Bacteroidetes* (100)** | unclassified | 0.778 | 0.571 | 0.008 |
|  | 128 | ***Cyanobacteria* (100)** | unclassified | 1.000 | 0.429 | 0.014 |
|  | 401 | ***Bacteroidetes* (100)** | ***Flavobacterium* (100)** | 1.000 | 0.429 | 0.019 |
|  | 250 | ***Cyanobacteria* (99)** | unclassified | 1.000 | 0.429 | 0.022 |
|  | 521 | ***Proteobacteria* (100)** | GKS98 freshwater group (97) | 1.000 | 0.429 | 0.012 |
|  | 918 | ***Planctomycetes* (100)** | ***Phycisphaera* (100)** | 1.000 | 0.429 | 0.015 |
|  | 731 | ***Proteobacteria* (100)** | ***Deefgea* (100)** | 1.000 | 0.429 | 0.018 |
|  | 752 | ***Verrucomicrobia* (100)** | ***Haloferula* (98)** | 1.000 | 0.429 | 0.023 |
|  | 1076 | ***Bacteroidetes* (100)** | ***Rheinheimera* (99)** | 1.000 | 0.429 | 0.017 |
|  | 1455 | ***Firmicutes* (91)** | Erysipelotrichaceae UCG-004 (71) | 1.000 | 0.429 | 0.018 |
|  | 1058 | ***Bacteroidetes* (100)** | ***Ferruginibacter* (100)** | 1.000 | 0.429 | 0.014 |
|  | 1071 | ***Proteobacteria* (100)** | ***Pseudospirillum* (100)** | 1.000 | 0.429 | 0.020 |
|  | 1114 | *Proteobacteria* (72) | *Fretibacter* (6) | 1.000 | 0.429 | 0.013 |
|  | 111 | ***Bacteroidetes* (100)** | ***Flavobacterium* (100)** | 0.976 | 0.429 | 0.030 |
|  | 1075 | ***Bacteroidetes* (100)** | ***Pedobacter* (97)** | 0.727 | 0.571 | 0.019 |
|  | 197 | ***Chloroflexi* (100)** | ***Roseiflexus* (100)** | 0.955 | 0.429 | 0.024 |
|  | 210 | ***Verrucomicrobia* (98)** | unclassified | 0.952 | 0.429 | 0.031 |
|  | 692 | ***Armatimonadetes* (100)** | ***Armatimonas* (100)** | 0.923 | 0.429 | 0.021 |
|  | 983 | ***Proteobacteria* (100)** | *Rhodobacter* (23) | 0.778 | 0.429 | 0.046 |
|  |  |  |  |  |  |  |
| PWs | 55 | ***Cyanobacteria* (100)** | unclassified | 0.827 | 0.571 | 0.033 |
|  | 49 | ***Cyanobacteria* (89)** | unclassified | 0.942 | 0.429 | 0.036 |
|  | 603 | ***Proteobacteria* (100)** | *Shinella* (54) | 0.706 | 0.571 | 0.013 |
|  | 156 | ***Planctomycetes* (100)** | ***Singulisphaera* (92)** | 0.891 | 0.429 | 0.028 |
|  | 475 | ***Planctomycetes* (100)** | unclassified | 0.833 | 0.429 | 0.045 |
|  |  |  |  |  |  |  |
| HTWs | 18 | ***Proteobacteria* (100)** | ***Novosphingobium* (92)** | 0.966 | 0.667 | 0.047 |
|  | 182 | ***Gemmatimonadetes* (98)** | unclassified | 0.933 | 0.500 | 0.004 |
|  | 338 | ***Proteobacteria* (100)** | ***Legionella* (100)** | 0.896 | 0.500 | 0.019 |
|  | 104 | ***Proteobacteria* (100)** | *Blastochloris* (28) | 0.870 | 0.500 | 0.027 |
|  | 309 | ***Proteobacteria* (100)** | unclassified | 0.757 | 0.500 | 0.016 |
|  | 217 | ***Proteobacteria* (100)** | *Ponticaulis* (49) | 0.752 | 0.500 | 0.032 |
|  | 343 | ***Proteobacteria* (100)** | unclassified | 0.739 | 0.500 | 0.030 |
|  | 352 | ***Proteobacteria* (100)** | unclassified | 0.724 | 0.500 | 0.027 |
|  | 375 | ***Proteobacteria* (100)** | unclassified | 1.000 | 0.333 | 0.027 |
|  | 886 | ***Omnitrophica* (98)** | unclassified | 1.000 | 0.333 | 0.027 |
|  | 553 | ***Planctomycetes* (100)** | unclassified | 1.000 | 0.333 | 0.027 |
|  | 1471 | *Proteobacteria* (70) | unclassified | 1.000 | 0.333 | 0.027 |
|  | 915 | ***Proteobacteria* (82)** | unclassified | 1.000 | 0.333 | 0.017 |
|  | 1174 | ***Planctomycetes* (89)** | unclassified | 1.000 | 0.333 | 0.027 |
|  | 196 | ***Planctomycetes* (100)** | ***Planctomyces* (99)** | 0.724 | 0.333 | 0.034 |

^a^ GW, groundwater; SW, surface water; PW, processed water; and HTW, household tap water; g or s, originating from groundwater or surface water, respectively.

^b^ Phylum identification based on the Silva database; bootstrap confidence values are given within parentheses; taxonomic assignments are generally considered reliable when bootstrap confidence values exceed 80 (indicated in bold).

^c^ Genus identification based on the Silva database; bootstrap confidence values are given within parentheses; taxonomic assignments are generally considered reliable when bootstrap confidence values exceed 80 (indicated in bold); when a confidence value of 0 was obtained, the OTU is considered “unclassified”.

^d^ Specificity score between 0 and 1; the closer to 1, the more the OTU is a robust indicator for the water type (a score of 1 indicates a unique OTU).

^e^ Fidelity score between 0 and 1; the higher the score the more samples within the group contain that OTU (a score of 1 indicates that all samples of the group contain the OTU).

^f^ OTUs with a *p* value less than 0.05 are considered significant indicators.

Table S5: Results of indicator species analysis for the two different sampling periods (April and November).

| **Sampling period** | **OTU ID** | **Phylum^a^** | **Genus^b^** | **A^c^** | **B^d^** | ***p* value^e^** |
| --- | --- | --- | --- | --- | --- | --- |
| April | 1 | ***Proteobacteria* (100)** | ***Acinetobacter* (100)** | 0.992 | 0.667 | 0.004 |
|  | 122 | ***Proteobacteria* (100)** | ***Vibrio* (100)** | 0.966 | 0.476 | 0.003 |
|  | 62 | ***Proteobacteria* (100)** | ***Stenotrophomonas* (98)** | 1.000 | 0.429 | 0.002 |
|  | 91 | ***Proteobacteria* (100)** | unclassified | 0.816 | 0.524 | 0.018 |
|  | 430 | ***Proteobacteria* (100)** | *Klebsiella* (46) | 0.937 | 0.429 | 0.007 |
|  | 84 | ***Cyanobacteria* (100)** | unclassified | 1.000 | 0.381 | 0.001 |
|  | 34 | ***Bacteroidetes* (100)** | ***Flavobacterium* (100)** | 0.982 | 0.381 | 0.023 |
|  | 160 | ***Proteobacteria* (100)** | *Thioalkalispira* (63) | 0.977 | 0.381 | 0.008 |
|  | 239 | ***Firmicutes* (100)** | ***Brevibacillus* (100)** | 0.942 | 0.381 | 0.009 |
|  | 246 | ***Bacteroidetes* (100)** | ***Chryseobacterium* (99)** | 1.000 | 0.333 | 0.007 |
|  | 115 | ***Bacteroidetes* (100)** | ***Hydrotalea* (99)** | 1.000 | 0.286 | 0.023 |
|  | 487 | ***Cyanobacteria* (100)** | unclassified | 1.000 | 0.286 | 0.026 |
|  | 208 | ***Proteobacteria* (100)** | ***Sphingopyxis* (100)** | 0.938 | 0.286 | 0.022 |
|  | 238 | ***Proteobacteria* (100)** | ***Novosphingobium* (84)** | 0.935 | 0.286 | 0.047 |
|  | 369 | ***Proteobacteria* (100)** | unclassified | 1.000 | 0.238 | 0.036 |
|  | 492 | ***Proteobacteria* (100)** | ***Pseudomonas* (88)** | 1.000 | 0.238 | 0.049 |
|  | 742 | ***Nitrospirae* (100)** | unclassified | 1.000 | 0.238 | 0.036 |
|  | 264 | ***Bacteroidetes* (100)** | ***Sphingobacterium* (100)** | 1.000 | 0.238 | 0.044 |
|  |  |  |  |  |  |  |
| November | 10 | ***Firmicutes* (100)** | *Bhargavaea* (24) | 0.764 | 0.900 | 0.001 |
|  | 24 | ***Actinobacteria* (100)** | ***Streptomyces* (82)** | 0.879 | 0.750 | 0.001 |
|  | 65 | ***Actinobacteria* (100)** | unclassified | 0.794 | 0.750 | 0.003 |
|  | 73 | ***Firmicutes* (100)** | ***Tumebacillus* (100)** | 0.850 | 0.700 | 0.002 |
|  | 8 | ***Proteobacteria* (100)** | ***Mizugakiibacter* (100)** | 0.896 | 0.650 | 0.002 |
|  | 172 | ***Actinobacteria* (100)** | ***Streptomyces* (100)** | 0.802 | 0.700 | 0.009 |
|  | 275 | ***Chloroflexi* (100)** | unclassified | 1.000 | 0.550 | 0.001 |
|  | 194 | ***Proteobacteria* (100)** | ***Sphingomonas* (97)** | 0.816 | 0.650 | 0.004 |
|  | 912 | ***Actinobacteria* (100)** | ***Streptacidiphilus* (96)** | 0.814 | 0.650 | 0.006 |
|  | 165 | ***Bacteroidetes* (100)** | *Flavisolibacter* (48) | 0.738 | 0.700 | 0.003 |
|  | 50 | ***Proteobacteria* (100)** | ***Sulfuricurvum* (100)** | 0.996 | 0.500 | 0.001 |
|  | 31 | ***Gemmatimonadetes* (100)** | unclassified | 0.993 | 0.500 | 0.001 |
|  | 163 | ***Acidobacteria* (100)** | ***Bryobacter* (98)** | 0.747 | 0.650 | 0.005 |
|  | 42 | ***Acidobacteria* (100)** | ***Acidobacterium* (100)** | 0.766 | 0.600 | 0.005 |
|  | 11 | ***Proteobacteria* (100)** | unclassified | 0.917 | 0.500 | 0.013 |
|  | 5 | ***Proteobacteria* (100)** | ***Phreatobacter* (100)** | 0.913 | 0.500 | 0.029 |
|  | 192 | ***Proteobacteria* (100)** | ***Sphingomonas* (100)** | 0.820 | 0.550 | 0.008 |
|  | 420 | ***Planctomycetes* (100)** | ***Schlesneria* (100)** | 0.973 | 0.450 | 0.002 |
|  | 679 | ***Chloroflexi* (96)** | unclassified | 0.957 | 0.450 | 0.003 |
|  | 222 | ***Actinobacteria* (100)** | *Micromonospora* (56) | 0.766 | 0.550 | 0.009 |
|  | 46 | ***Actinobacteria* (100)** | unclassified | 0.697 | 0.600 | 0.024 |
|  | 350 | ***Actinobacteria* (100)** | unclassified | 0.759 | 0.550 | 0.014 |
|  | 7 | ***Proteobacteria* (100)** | ***Gallionella* (97)** | 0.920 | 0.450 | 0.034 |
|  | 90 | ***Actinobacteria* (100)** | ***Blastococcus* (100)** | 1.000 | 0.400 | 0.001 |
|  | 82 | ***Proteobacteria* (100)** | ***Ralstonia* (100)** | 0.988 | 0.400 | 0.004 |
|  | 493 | ***Actinobacteria* (100)** | unclassified | 0.876 | 0.450 | 0.013 |
|  | 107 | ***Chloroflexi* (100)** | unclassified | 0.982 | 0.400 | 0.002 |
|  | 519 | ***Firmicutes* (100)** | ***Tumebacillus* (100)** | 0.856 | 0.450 | 0.006 |
|  | 99 | ***Proteobacteria* (100)** | ***Mizugakiibacter* (100)** | 0.944 | 0.400 | 0.016 |
|  | 380 | ***Proteobacteria* (100)** | ***Devosia* (92)** | 0.817 | 0.450 | 0.025 |
|  | 992 | ***Proteobacteria* (100)** | ***Gallionella* (94)** | 0.897 | 0.400 | 0.006 |
|  | 146 | ***Actinobacteria* (100)** | ***Patulibacter* (96)** | 0.796 | 0.450 | 0.017 |
|  | 150 | ***Cyanobacteria* (100)** | unclassified | 1.000 | 0.350 | 0.003 |
|  | 437 | ***Proteobacteria* (100)** | *Crenothrix* (61) | 0.869 | 0.400 | 0.044 |
|  | 747 | ***Gemmatimonadetes* (100)** | unclassified | 0.869 | 0.400 | 0.030 |
|  | 301 | ***Actinobacteria* (100)** | unclassified | 0.963 | 0.350 | 0.003 |
|  | 77 | ***Gemmatimonadetes* (100)** | unclassified | 0.920 | 0.350 | 0.027 |
|  | 322 | ***Acidobacteria* (100)** | *Acidobacterium* (76) | 0.913 | 0.350 | 0.025 |
|  | 289 | ***Actinobacteria* (100)** | ***Actinomadura* (100)** | 0.798 | 0.400 | 0.050 |
|  | 653 | ***Actinobacteria* (100)** | unclassified | 0.904 | 0.350 | 0.014 |
|  | 377 | ***Firmicutes* (100)** | ***Bacillus* (83)** | 0.894 | 0.350 | 0.040 |
|  | 600 | ***Proteobacteria* (100)** | ***Microvirga* (100)** | 0.880 | 0.350 | 0.039 |
|  | 201 | ***Proteobacteria* (100)** | unclassified | 0.876 | 0.350 | 0.048 |
|  | 501 | ***Gemmatimonadetes* (100)** | ***Gemmatimonas* (100)** | 1.000 | 0.300 | 0.009 |
|  | 586 | *Euryarchaeota* (40) | unclassified | 1.000 | 0.300 | 0.015 |
|  | 262 | ***Proteobacteria* (100)** | *Thermomonas* (70) | 0.825 | 0.350 | 0.032 |
|  | 764 | ***Actinobacteria* (100)** | ***Modestobacter* (100)** | 0.944 | 0.300 | 0.030 |
|  | 885 | ***Gemmatimonadetes* (100)** | *Gemmatimonas* (53) | 0.794 | 0.350 | 0.040 |
|  | 686 | ***Chloroflexi* (100)** | ***Sphaerobacter* (95)** | 0.904 | 0.300 | 0.026 |
|  | 879 | ***Firmicutes* (100)** | *Ammoniphilus* (63) | 0.904 | 0.300 | 0.026 |
|  | 274 | ***Actinobacteria* (100)** | *Actinoplanes* (48) | 1.000 | 0.250 | 0.023 |
|  | 363 | **Candidate division OP3 (100)** | unclassified | 1.000 | 0.250 | 0.017 |
|  | 523 | ***Actinobacteria* (100)** | ***Acidothermus* (98)** | 1.000 | 0.250 | 0.025 |
|  | 705 | ***Actinobacteria* (100)** | unclassified | 1.000 | 0.250 | 0.023 |
|  | 136 | ***Acidobacteria* (100)** | unclassified | 1.000 | 0.250 | 0.019 |
|  | 931 | ***Actinobacteria* (100)** | *Jatrophihabitans* (22) | 1.000 | 0.250 | 0.020 |
|  | 1112 | ***Proteobacteria* (100)** | *Oceanicoccus* (19) | 1.000 | 0.250 | 0.022 |
|  | 1382 | ***Parcubacteria* (100)** | unclassified | 1.000 | 0.250 | 0.020 |
|  | 87 | ***Actinobacteria* (100)** | **hgcI clade (100)** | 0.940 | 0.250 | 0.038 |
|  | 479 | ***Proteobacteria* (100)** | ***Skermanella* (100)** | 1.000 | 0.200 | 0.040 |
|  | 308 | ***Bacteroidetes* (100)** | *Leadbetterella* (100) | 1.000 | 0.200 | 0.050 |
|  | 450 | ***Bacteroidetes* (100)** | unclassified | 1.000 | 0.200 | 0.050 |
|  | 481 | ***Actinobacteria* (100)** | ***Crossiella* (100)** | 1.000 | 0.200 | 0.047 |
|  | 893 | ***Firmicutes* (100)** | *Bacillus* (23) | 1.000 | 0.200 | 0.044 |
|  | 631 | ***Firmicutes* (100)** | *Thalassobacillus* (46) | 1.000 | 0.200 | 0.050 |
|  | 957 | ***Proteobacteria* (100)** | unclassified | 1.000 | 0.200 | 0.044 |
|  | 1119 | ***Proteobacteria* (100)** | Candidatus Gigarickettsia (32) | 1.000 | 0.200 | 0.048 |
|  | 1306 | ***Firmicutes* (100)** | ***Terrisporobacter* (100)** | 1.000 | 0.200 | 0.042 |
|  | 844 | ***Proteobacteria* (100)** | unclassified | 1.000 | 0.200 | 0.042 |
|  | 890 | ***Firmicutes* (100)** | ***Peptoclostridium* (93)** | 1.000 | 0.200 | 0.042 |

^a^ Phylum identification based on the Silva database; bootstrap confidence values are given within parentheses; taxonomic assignments are generally considered reliable when bootstrap confidence values exceed 80 (indicated in bold).

^b^ Genus identification based on the Silva database; bootstrap confidence values are given within parentheses; taxonomic assignments are generally considered reliable when bootstrap confidence values exceed 80 (indicated in bold); when a confidence value of 0 was obtained, the OTU is considered “unclassified”.

^c^ Specificity score between 0 and 1; the closer to 1, the more the OTU is a robust indicator for the water type (a score of 1 indicates a unique OTU).

^d^ Fidelity score between 0 and 1; the higher the score the more samples within the group contain that OTU (a score of 1 indicates that all samples of the group contain the OTU).

^e^ OTUs with a *p* value less than 0.05 are considered significant indicators.

Figure S1: Rarefaction curves generated for each individual water sample. These curves illustrate the accumulated number of bacterial Operational Taxonomic Units (OTUs) based on a DNA dissimilarity cut-off value of 3 %. Brown lines represent surface water, red lines groundwater, blue lines processed water, and orange lines household tap water. For more information about the diversity measures for the individual samples the reader is referred to Table S1 (Supporting Information).

GW, PWg

and HTWg

April

November

SW, PWs

and HTWs

120

100

80

60

40

20

0

120

100

80

60

40

20

0

Water type

Sampling period

Number of sequences

Number of sequences

**Figure S2:** Boxplot representation of the number of *Nitrospirae* sequences in the water samples investigated in this study. Water samples were grouped based on water type (**A**) and sampling period (**B**). The boxplots show the upper and lower quartiles; the whiskers indicate variability outside the upper and lower quartiles which is no more than 1.5 times the interquartile range. Further, the median is plotted as a thick black line. GW, PWg and HTWg, groundwater, processed water produced from groundwater and household tap water processed from groundwater (*n* =21); SW, PWs and HTWs, surface water, processed water produced from surface water and household tap water processed from surface water (*n* = 20); April (*n* = 21); November (*n* = 20).

**Figure S3:** Heatmap representation showing the differences in bacterial community composition of the water samples investigated in this study (columns represent different OTUs). A Bray-Curtis distance matrix was used and the samples were clustered by a UPGMA agglomeration method. GW, groundwater; PWg, processed water originating from groundwater; HTWg, household tap water originating from groundwater; SW, surface water; PWs, processed water originating from surface water; HTWs, household tap water originating from surface water. For more information about the studied samples the reader is referred to Table S1 (Supporting Information)

**Figure S4:** Rooted neighbor-joining tree based on the V4 region of the 16S ribosomal RNA gene (250 bp), positioning unique sequences of the three *Acinetobacter* OTUs identified in this study (OTU1, OTU293 and OUT 1434) among reference sequences of all known *Acinetobacter* species and a number of *Acinetobacter* genomic species. In total 11 clusters can be observed housing *Acinetobacter* sequences recovered in this study.
